# Dissection of a major QTL *qhir1* conferring maternal haploid induction ability in maize

**DOI:** 10.1007/s00122-017-2873-9

**Published:** 2017-03-18

**Authors:** Sudha K. Nair, Willem Molenaar, Albrecht E. Melchinger, Prasanna M. Boddupalli, Leocadio Martinez, Luis Antonio Lopez, Vijay Chaikam

**Affiliations:** 1 International Maize and Wheat Improvement Center (CIMMYT), ICRISAT campus, Patancheru, Greater Hyderabad, 502324 India; 2 Institute of Plant Breeding, Seed Science and Population Genetics, University of Hohenheim, 70593 Stuttgart, Germany; 3 International Maize and Wheat Improvement Center (CIMMYT), ICRAF campus, UN Avenue, Gigiri, P.O.Box 1041–00621, Nairobi, Kenya; 4 International Maize and Wheat Improvement Center (CIMMYT), Apdo. Postal 6-641, 06600 Mexico, D.F Mexico

## Abstract

**Key message:**

Among the *qhir11* and *qhir12* sub-regions of a major QTL *qhir1*, only *qhir11* has significant effect on maternal haploid induction, segregation distortion and kernel abortion.

**Abstract:**

In vivo haploid induction in maize can be triggered in high frequencies by pollination with special genetic stocks called haploid inducers. Several genetic studies with segregating populations from non-inducer x inducer crosses identified a major QTL, *qhir1*, on chromosome 1.04 contributing to in vivo haploid induction. A recent Genome Wide Association Study using 51 inducers and 1482 non-inducers also identified two sub-regions within the *qhir1* QTL region, named *qhir11* and *qhir12; qhir12* was proposed to be mandatory for haploid induction because the haplotype of *qhir11* was also present in some non-inducers and putative candidate genes coding for DNA and amino acid binding proteins were identified in the *qhir12* region. To characterize the effects of each sub-region of *qhir1* on haploid induction rate, F_2_ recombinants segregating for one of the sub-regions and fixed for the other were identified in a cross between CML269 (non-inducer) and a tropicalized haploid inducer TAIL8. To quantify the haploid induction effects of *qhir11* and *qhir12*, selfed progenies of recombinants between these sub-regions were genotyped. F_3_ plants homozygous for *qhir11* and/or *qhir12* were identified, and crossed to a *ligueless* tester to determine their haploid induction rates. The study revealed that only the *qhir11* sub-region has a significant effect on haploid induction ability, besides causing significant segregation distortion and kernel abortion, traits that are strongly associated with maternal haploid induction. The results presented in this study can guide fine mapping efforts of *qhir1* and in developing new inducers efficiently using marker assisted selection.

**Electronic supplementary material:**

The online version of this article (doi:10.1007/s00122-017-2873-9) contains supplementary material, which is available to authorized users.

## Introduction

Large-scale production and utilization of doubled haploid (DH) lines has become common practice in maize breeding programs during the last decade owing to the associated acceleration and cost reduction in development of inbred lines and deployment of hybrid varieties (Melchinger et al. 2013). In vivo maternal haploid induction (HI) is the backbone of DH line production in maize (Prigge et al. 2012b), which involves pollination of desired populations with special genetic stocks called haploid inducers that induce relatively high frequencies of haploid seeds in the progeny (Coe 1959; Chaikam 2012; Prigge and Melchinger 2012). The phenomenon of in vivo maternal HI is unique to maize and has not been reported in other plant species so far (Hu et al. 2016), although its physiological and molecular bases are still elusive. Elimination of inducer chromosomes after fertilization (Zhang et al. 2008; Li et al. 2009; Xu et al. 2013a; Qiu et al. 2014) and single fertilization followed by parthenogenesis (Sarkar and Coe 1966; Bylich and Chalyk 1996; Barret et al. 2008; Swapna and Sarkar 2012) were proposed to be involved in the production of seeds with haploid embryos and normal triploid endosperms.

To understand the genetic basis of HI, several studies have been conducted. HI was determined to be a quantitatively inherited trait, controlled by a small number of genes and improvable through selection (Lashermes and Beckert 1988). It was also suggested that additive and epistatic gene action affect the HI process (Prigge et al. 2011). In first QTL mapping studies on HI with segregating progeny of crosses between non-inducers and inducers, a major QTL on chromosome 1 was identified in bin 1.04 (Deimling et al. 1997; Barret et al. 2008). An extensive QTL mapping study with four bi-parental populations involving inducers CAUHOI and UH400 detected two major QTL, named *qhir1* and *qhir8*, and several minor QTL (Prigge et al. 2012b). The major QTL *qhir1* on chromosome 1.04 was the same as reported in the previous studies and explained 66% of the genotypic variance. Besides its effect on HI, *qhir1* has also been associated with segregation distortion (SD) and has a strong selective disadvantage (Barret et al. 2008; Prigge et al. 2012b; Dong et al. 2013; Xu et al. 2013a). It was also noted that in vivo HI is associated with embryo and endosperm abortion (Prigge et al. 2012b; Xu et al. 2013a). Less pronounced than the effect of *qhir1* was the effect of the second major QTL found by Prigge et al. (2012b), *qhir8*, which maps to chromosome 9 and explained only 20% of the genotypic variance. However, all these linkage mapping studies resulted in large support intervals for the detected QTL.

To delineate the map position and to identify closely linked markers more useful for marker-assisted selection in development of new inducers, *qhir1* was fine-mapped to a 243 kb region (Dong et al. 2013) and *qhir8* to a 789 kb region (Liu et al. 2015). Considering the confirmation of *qhir1* in multiple studies, *qhir1* may be considered mandatory for HI ability (Prigge et al. 2012b), while other loci like *qhir8* may enhance the function of *qhir1* to increase the HIR (Liu et al. 2015).

Recently, the large *qhir1* support interval described by Prigge et al. (2012b) was dissected by Hu et al. (2016) into two closely linked regions, named *qhir11* and *qhir12*, using a novel type of genome wide association study (GWAS) to detect selective sweeps and address the problem of perfect confounding between population structure and trait expression, as in the case of inducers (cases) and non-inducer (controls). Sub-region *qhir11* harbored the 243 kb interval fine-mapped by Dong et al. (2013) and had one major haplotype present in the majority of the inducers and one minor haplotype present only in two inducers studied. The latter occurred also in several non-inducers whose HIR was similar to spontaneous occurrence of haploids. Hence, the minor haplotype of *qhir11* was deemed to be neither diagnostic for differentiating inducers and non-inducers nor effective for conditioning HI ability in maize. However, no conclusions were drawn about the major haplotype of *qhir11* based on this study. By comparison, *qhir12* had a single haplotype allele found in all the 53 inducers and absent in all 1482 non-inducers included in the study and was proposed to harbor three candidate genes related to putative functions involved in HI. To further determine the effects of the *qhir12* and *qhir11* haplotypes, the authors suggested testing the effect of these haplotypes on HI in near-isogenic lines or selfed progenies of recombinants that segregate at one locus while the other is fixed.

The main objective of our study is to adopt this strategy and test the effects of *qhir11* and *qhir12* haplotypes on HIR using selfed progenies of recombinants in a huge F_2_ population derived by crossing a non-inducer with a tropically adapted haploid inducer. In addition, we examined which of the specific sub-regions of *qhir1* is specifically associated with segregation distortion and kernel abortion, traits associated with maternal haploid induction.

## Materials and methods

### Notation of the genotypes

We denote henceforth the *qhir11* and *qhir12* sub-regions as A and B, respectively. We use the following notations for the various genotypes possible for each sub-region: AA = homozygous for the putative inducer allele at all markers assayed in the *qhir11* sub-region; aa = homozygous for the putative non-inducer allele at all markers in the *qhir11* sub-region; BB = homozygous for the putative inducer allele at all markers assayed in the *qhir12* sub-region; bb = homozygous for the putative non-inducer allele at all markers in the *qhir12* sub-region; Aa = heterozygous at all markers assayed in the *qhir11* sub-region; and Bb = heterozygous for all markers assayed in the *qhir12* sub-region.

### Genetic material

One tropically adapted inducer, TAIL8, and one tropically adapted non-inducer, CML269, were used as parents in this study. TAIL8, harboring the A and B alleles in homozygous state has a mean HIR of 9.9% (Chaikam et al. 2016). CML269 has no HI ability and harbors the a and b alleles in homozygous state. The non-inducer (CML269) x inducer (TAIL8) cross was made in the winter season of 2011 at CIMMYT’s experimental station at Agua Fria, Mexico (20.26°N, 97.38°W) to generate the F_1_ generation. From the F_1_, 100 seeds were planted and selfed to generate the F_2_ generation in the summer season of 2011. A total of 7160 F_2_ seeds of good quality were genotyped as described below. Recombinants between the *qhir11* and *qhir12* sub-regions identified on the basis of the marker assays were grouped into four F_2_ genotype classes: AABb; aaBb, AaBB, Aabb, and used for further assays.

**Fig. 1:**
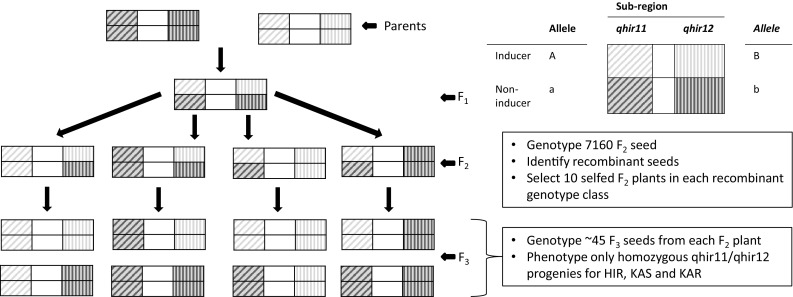
Strategy used for genetic delineation of *qhir1* and analysis of the effects of *qhir11* and *qhir12* sub-regions on HIR in maize

From each of the four F_2_ genotype classes of recombinants, 10 individual plants were randomly selected for selfing to obtain F_2:3_ families segregating for the heterozygous sub-region. Only F_3_ seeds homozygous for the segregating sub-region were planted in the field at Agua Fria in the winter season of 2016. Hybrid (PDH3 × PDH8), homozygous for *liguleless* gene *lg2* (Prigge et al. 2012a; Chaikam et al. 2016; Melchinger et al. 2016), was used as a female tester to produce testcross seed for evaluating the HIR. The tester was stagger-planted four times at weekly intervals to synchronize flowering with the F_3_ plants. Each F_3_ plant that produced pollen was crossed on to 10–15 tester plants (based on pollen availability) and was also self-pollinated. Some F_3_ plants were found to be haploids based on their weak plant stature, narrow and erect leaves and sterile tassels (Prigge et al. 2011; Chaikam et al. 2016) and were therefore not pollinated. Some plants could not be used for testcrossing because of severe virus infection. Testcross seed was bulked from all the tester plants pollinated by the same F_3_ plant. A graphical representation of the scheme followed for developing the genetic material is shown in Fig. 1.

### Markers delineating the *qhir11* and *qhir12* sub-regions

According to Hu et al. (2016), the physical boundaries for *qhir11* are between SNPs PZE-101,081,177 (physical co-ordinate: 1: 68,134,633) and SYN25793 (physical co-ordinate 1: 68,670,617). For *qhir12*, the borders are between SYN4966 (physical co-ordinate 1: 71,795,509) and PZA00714.1 (physical co-ordinate 1: 75,768,235). All the physical co-ordinates of the SNPs assayed are with reference to B73 AGP V2 (http://ensembl.gramene.org/Zea_mays). Sets of six markers covering the *qhir11* sub-region and eight markers covering the *qhir12* sub-region were used to genotype each sub-region (Supplementary table 1). Based on the selected SNPs, the haplotypes of TAIL8 and CML269 at each sub-region were compared with the large set of non-inducers and inducers reported by Hu et al. (2016) and verified. All markers used in this study were genotyped using KASP assays (LGC Genomics, UK) developed from the Illumina MaizeSNP50 BeadChip (Ganal et al. 2011), except for one SNP developed from HapMap V.2 (Suppl. Table 1).

### Analysis of the F_2_ population

DNA was extracted from 7160 individual seeds of the F_2_ population of cross TAIL8 × CML269 following standard procedures (Gao et al. 2008) and genotyped with the above-mentioned SNPs. Among the polymorphic SNPs between the two parents available to CIMMYT for the *qhir11* and *qhir12* sub-regions, two SNPs (PZE0166290049 and PZE0166357949) were selected to represent *qhir11* and two SNPs (SYN26730 and PZE101085336) to represent *qhir12* in the genotyping of the F_2_ seeds. Based on the results, 428 recombinant F_2_ seeds in the four F_2_ genotype classes described above were selected and planted in the field. Leaf DNA of these plants was extracted at the four-leaf stage following CIMMYT’s laboratory protocols (CIMMYT 2001). Because the two SNPs of each sub-region in the seeds did not cover the respective physical interval entirely, we analyzed additionally four SNPs for *qhir11* and 10 SNPs for *qhir12*, which were part of the SNPs on the MaizeSNP50 BeadChip polymorphic between the two parents. This assay was also used to ascertain the classification of the recombinant F_2_ plants; plants showing any discrepancy were discarded. Moreover, some F_2_ plants did not survive or failed to produce selfed seed. Thus, selfed ears were harvested from 21 AABb, 72 aaBb, 56 AaBB, and 44 Aabb genotypes in the F_2_ generation, adding up to a total of 193 ears.

### Genotyping and phenotypic analysis of F_2:3_ families from recombinants

Ten ears were randomly selected from each of the four afore-mentioned F_2_ genotype classes for raising F_2:3_ families. DNA was extracted from ~45 individual seeds from each of the 40 F_2:3_ families and genotyped with three SNP markers for both *qhir11* and *qhir12*, covering the entire physical interval of the sub-regions as identified by Hu et al. (2016). From each family, only the seeds homozygous for the *qhir11* and *qhir12* sub-regions were selected as male parents for pollination of *liguleless* tester PDH3 × PDH8.

Among the 756 F3 plants that were test-crossed, 83.7% resulted in more than 1000 seeds, 12% resulted between 500 and 999 seeds, and 4.2% resulted in less than 500 seeds. For each F_3_ plant with more than 1000 testcross seeds, 1000 seeds were germinated in styrofoam trays in a shade house at the Agua Fria experimental station. Each tray accommodated 100 seeds. After 14 days of germination, each tray was evaluated for the number of germinated seedlings and the number of seedlings with and without ligule. For the F_3_ plants with less than 1000 testcross seeds, all seeds were germinated. The HIR was calculated as HIR = *N*_L_/(*N*_L_+*N*_N*L*_), where *N*_L_ and *N*_NL_ refer to the number of plants with and without ligule, respectively.

### Phenotyping of kernel abortion

We refer here to endosperm abortion as kernel abortion, because most endosperm aborted seeds in our study lacked an embryo similar to the observation by Xu et al. (2013a). Selfed ears obtained from the F_3_ plants in each of the four genotype classes were visually rated for a kernel abortion score (KAS) on a scale of 1–5, where 1 represents no aborted seed visible on the ear, and 5 represents complete abortion with no seed set on the ear. To measure the extent of kernel abortion quantitatively, the number of normal seeds and number of kernel aborted seeds were counted on each ear from the AAbb and aaBB genotype classes as suggested by Xu et al. (2013a). Kernel abortion rate (KAR) for each entry was calculated as KAR = *N*_a_/(*N*_a_ + *N*_n_), where *N*_a_ refers to the number of aborted seeds and *N*_n_ to the number of normal seeds.

### Statistical analyses

The HIR for each F_3_ genotype within each F_2_ genotype class was calculated as the least-squares means in the following generalized linear model assuming a binomial distribution:

$${{Y}_{ijk}}=\text{ }\mu \text{ }+\text{ }{{g}_{j~}}+\text{ }{{f}_{k}}_{{}}+\text{ }{{e}_{ijk}},$$

where *Y*_*ijk*_ is the *i*th observation in the *j*th genotype class for the *k*th F_2:3_ family, *µ* is the general mean, *g*_*j*_ is the effect of the *j*th genotype, *f*_*k*_ is the effect of the *k*th family and *e*_*ijk*_ the residual error. The model was fitted using the *glm* function in the R software package, version 3.3.0. Least-squares means and corresponding confidence intervals were calculated with the *lsmeans* package, version 2.23–5, and compact letters displays were produced with the *multcompView* package, version 0.1–7, at significance level *α* = 5%. We used an over-dispersion factor to account for variance in the data in excess of the binomial sampling variance that may result in an inflation of the standard errors.

For each of the four recombinant genotype classes selected from the F_2_, we tested the following hypotheses: (i) H_0_: $\bar{g}$_aabb_ = $\bar{g}$_AAbb_ vs. H_A_: $\bar{g}$_aabb_ ≠ $\bar{g}$_AAbb_ (from F_2_ genotype class Aabb); (ii) H_0_: $\bar{g}$_aabb_ = $\bar{g}$_aaBB_ vs. H_A_: $\bar{g}$_aabb_ ≠ $\bar{g}$_aaBB_ (from F_2_ genotype class aaBb); (iii) H_0_: $\bar{g}$_AABB_ = $\bar{g}$_aaBB_ vs. H_A_: $\bar{g}$_AABB_ ≠ $\bar{g}$_aaBB_ (from F_2_ genotype class AaBB); and (iv) H_0_: $\bar{g}$_AABB_ = $\bar{g}$_AAbb_ vs. H_A_: $\bar{g}$_AABB_ ≠ $\bar{g}$_AAbb_ (from F_2_ genotype class AABb).

F_3_ plants heterozygous for either of the sub-regions were not tested in this experiment. Significant differences in these tests determine whether the *qhir11* or *qhir12* sub-region alone is sufficient to exhibit HIR equivalent to *qhir11* and *qhir12* together.

KAS for each F_3_ genotype was calculated with the same generalized linear model as for HIR except that a Poisson distribution was assumed and KAS was used as response variable. KAR for the two F_3_ genotype classes AAbb and aaBB was calculated with the same generalized linear model but without the family term because of confounding between family and genotype.

Segregation distortion (SD) in the F_2_ generation was investigated with a G-test for goodness-of-fit to the segregation ratios expected under Mendelian inheritance and applying a significance level *α* = 5%. The G-test of goodness of fit to expected segregation ratios and the expected allele frequencies was carried out with the R software function *GTest* from the *DescTools* package, version 0.99.17.

## Gene annotations

The gene annotations by the MAKER gene annotation pipeline (Cantarel et al. 2008) in the physical interval of *qhir11* in the B73 genome sequence (V2) available in http://ensembl.gramene.org/Zea_mays was used to search for putative candidate genes in the studied interval.

## Results

### Recombination and segregation in the F_2_ and F_3_ generations

A total of 475 recombinants in the F_2_ generation falling into different genotype classes were identified between *qhir11* and *qhir12* based on the segregation analysis of 7154 F_2_ seeds (Table 1). No recombination was observed between the two sub-regions in most of the F_2_ seeds (93.4%), which had the same genotype as the F_1_ cross or the parent lines. Single recombination events between *qhir11* and *qhir12* were observed in 6% of the F_2_ seeds, and double recombination events between *qhir11* and *qhir12* were observed in 0.1% of F_2_ seeds. In addition, 0.6% of F_2_ seeds had recombination events which occurred within either of the sub-regions. Based on the recombination observed between the distal SNP of *qhir11* and the proximal SNP of *qhir12*, the recombination rate between the *qhir11* and *qhir12* sub-regions was 3.1%. From the 428 single recombinant F_2_ plants between *qhir11* and *qhir12*, a total of 193 plants remained for further analyses, with the following numbers in the four F_2_ genotype classes: 72 aaBb, 56 AaBB, 44 Aabb and 21 AABb. Among F_3_ plants, highly significant (*P* < 0.001) segregation distortion against the homozygous inducer genotype was observed for the *qhir11* sub-region (Table 2). The segregation distortion observed for the *qhir12* sub-region was also significant (*P* < 0.01) but against the non-inducer genotype. The same trends were observed for the allele frequencies at both sub-regions.

**Table 1:** Recombination in parental gametes observed in the F_2_ generation between the sub-regions *qhir11* and *qhir12*

No. of recombinations	Genotype^a^	Counts	Frequency (%)
0	aabb	2340	32.7
	AABB	1020	14.25
	AaBb	3319	46.38
1 (*∑* = 428)	AABb	59	0.82
	aaBb	174	2.43
	AaBB	106	1.48
	Aabb	89	1.24
2 (*∑* = 6)	AAbb	2	0.03
	aaBB	4	0.06

^a^aa and bb (homozygous for the non-inducer CML269 in sub-region *qhir11* and *qhir12*, respectively, based on two SNPs assayed in each sub-region); AA and BB (homozygous for the inducer TAIL8); Aa and Bb heterozygous

**Table 2:** Segregation, allele counts and allele frequencies observed for sub-regions *qhir11* and *qhir12* in F_3_ seeds from the four F_2_ genotype classes segregating only at the respective locus. *P* values are shown for a G-test of goodness of fit of observed counts to the expected segregation ratio of 0.25:0.50:0.25, and for expected allele frequencies of 0.5

Sub-region	Genotypes in F_3_			Alleles in F_3_	
*qhir11*	AA	Aa	aa	A	a
Counts	150	455	323	755	1101
%	0.16	0.49	0.35	0.41	0.59
G-test	*P* = 7.42 × 10^− 15^			*P* = 8.03 × 10^− 16^	

*qhir12*	BB	Bb	aa	B	b
Counts	212	464	174	888	812
%	0.25	0.55	0.20	0.52	0.48
G-test	*P* = 0.00428			*P* = 0.0652	

### Effects of the *qhir11* and *qhir12* sub-regions on haploid induction rate

F_3_ plants with genotype AAbb, derived from F_2_ plants in genotype class Aabb, revealed on average a significantly (*P* < 0.01) higher HIR (6.45%) than aabb plants having a mean HIR = 0.12% (Table 3). Thus, the AA genotype showed a strong positive effect on HIR. In F_3_ plants of F_2_ genotype class AABb, HIR was significantly (*P* < 0.01) higher in AAbb plants (7.16%) than in AABB plants (5.92%). Thus, a relatively small negative effect on HIR was observed for the BB genotype in the presence of the AA genotype. This negative effect was not observed in the absence of the AA genotype, because in F_3_ plants from F_2_ genotype class aaBb, the mean HIR of the aaBB genotypes (0.12%) was not significantly different from the mean HIR of the aabb genotypes (0.09%). In F_3_ plants of the F_2_ genotype class AaBB, the HIR of AABB genotypes was also significantly (*P* < 0.01) higher than the HIR of aaBB genotypes.

**Table 3:** Least-squares means for HIR for each F_3_ genotype within each of the four F_2_ genotype classes

F_2_ genotype class	F_3_ genotype	No. seedlings		HIR%^a^
		Haploid	Diploid	
Aabb	aabb	156	132,126	0.12^a^
	AAbb	3770	52,658	6.45^b^
AaBB	aaBB	262	106,820	0.24^a^
	AABB	2889	46,736	5.94^b^
aaBb	aabb	87	87,819	0.09^a^
	aaBB	125	98,396	0.12^a^
AABb	AAbb	3933	51,616	7.16^a^
	AABB	4016	61,841	5.92^b^

^a^Different letters (a and b) indicate a significant difference in least-squares means of HIR between genotypes within each F_2_ genotype class at an overall significance level *α* = 5%, with a Bonferroni correction for multiple comparisons

Regarding the HIR of all F_3_ plants irrespective of their origin from the four F_2_ genotype classes, the highest HIR (5.96%) was observed for genotype AAbb, followed by a significantly (*P* < 0.05) smaller value (HIR = 5.02%) for genotype AABB (Table 4). A large decrease in HIR was found in the aaBB genotype (HIR = 0.19%) and a further significant (*P* < 0.05) decrease in the aabb genotype (HIR = 0.12%). Thus, in the presence of AA at *qhir11*, BB had a reducing effect on HIR but in the presence of aa, it had an increasing effect on HIR, whereas no significant effect was observed in the analysis of means in F_3_ genotypes derived from individual F_2_ genotype classes.

**Table 4:** Least-squares means for haploid induction rate (HIR), kernel abortion score (KAS) and kernel abortion rate (KAR) for each F_3_ genotype

Genotype	HIR^a^	KAS^ab^	KAR^ab^
aabb	0.12^a^	1.25^a^	–
aaBB	0.19^b^	1.34^a^	4.26^a^
AAbb	5.96^d^	2.99^b^	29.62^b^
AABB	5.02^c^	2.84^b^	–

^a^Different letters indicate significant differences at an overall significance level *α* = 5%, using the Tukey method for comparing a group of four estimates for HIR and KAS and no adjustment for KAR, because only one comparison could be made

^b^KAS is shown on the original score scale, KAR is shown in percent

### Effects of the *qhir11* and *qhir12* sub-regions on kernel abortion

Most ears harvested from AAbb and AABB genotypic class F3 plants showed some level of kernel abortion while most ears of aaBB and aabb classes did not record any abortion (Suppl. Figure 1a and 1b). Regardless of the genotype at the other sub-region, F_3_ plants of genotype AA had a significantly (*P* < 0.01) higher KAS than the aa genotype. Quantitative evaluation of kernel abortion in the F_3_ generation showed that genotype AAbb had a six-fold higher KAR than the genotype aaBB (Table 4).

## Discussion

### Strategy for genetic delineation of *qhir1* influencing haploid induction

Genetic delineation of the *qhir11* and *qhir12* sub-regions required large population sizes in the F_2_ generation considering that they are physically located very close to each other on chromosome 1.04 (Hu et al. 2016). Regarding the incomplete penetrance of *qhir1* for HIR (Prigge et al. 2012b), the choice of the parents for this study was critical to guarantee sufficient variation in HIR of progenies recombinant for the *qhir11* and *qhir12* sub-regions. The non-inducer parent CML269 had shown highly significant difference in HIR values between progeny selected for *qhir1* in combination with multiple haploid inducers (CIMMYT, unpublished data). Therefore, CML269 was chosen as non-inducer parent to develop a large F_2_ population with the selected tropicalized haploid inducer TAIL8. The 14 SNP markers selected for our analyses provided good coverage of the *qhir1* region and were sufficient to delineate the sub-regions *qhir11* and *qhir12*. The recombinants observed in the F_2_ generation showed a genetic distance of 3.1 cM between them, which is consistent with the estimate for *qhir11* and *qhir12* reported by Hu et al. (2016). We did not study the effect of *qhir11* and *qhir12* in homozygous recombinants (AAbb or aaBB) of the F_2_ generation because they were too few to make valid inferences. Given the huge efforts required in phenotyping for HIR, we had to restrict the number of individuals analyzed from each F_2_ genotype class to 10 F_2_ plants, resulting in 40 F_2:3_ families which could be analyzed within and among the four genotype classes. Seed DNA was genotyped for each of these F_3_ families to eliminate heterozygotes before planting and conducting testcrosses and selfings with the F_3_ plants. A *liguless* tester was used in testcrosses for measuring HIR because this method was recommended for accurate measurement of HIR in comparison to other methods (Melchinger et al. 2016) and has been reliably used for determining the HIR in previous studies (Prigge et al. 2012a; Melchinger et al. 2013; Chaikam et al. 2016). Staggered planting of the *liguleless* tester multiple times allowed achieving synchrony in flowering with the majority of the F_3_ plants differing widely in anthesis date (data not shown). For the majority of F_3_ plants (83.7%), we could evaluate HIR based on the recommended number of testcross seed (1000) and for only less than 1% of the F_3_ plants we had to measure HIR with fewer than 200 testcross seeds, which was the lower limit suggested by Prigge et al. (2012a).

### Effects of *qhir11* and *qhir12* on maternal haploid induction rate

The F_3_ progenies, which were homozygous recombinants for the *qhir11* and *qhir12* sub-region, showed unambiguous differences in HIR (Table 3). HIR is known to be a trait with incomplete penetrance and hence, has a tendency to show highly variable expression in different genetic backgrounds (Prigge et al. 2012b). In the population studied here, there appeared to be no alleles masking the HIR trait, because HIR ranged from normal inducer levels to non-inducer levels. In contrast to the hypothesis put forward by Hu et al. (2016), the 535 kb segment of the *qhir11* sub-region was in our study the only sub-region of *qhir1* mandatory for HI ability. The inducer *qhir11* allele (A) increased the HIR significantly in the presence of inducer (B) or non-inducer (b) alleles at the sub-region *qhir12*. The inducer *qhir12* allele alone, in the absence of the inducer *qhir11* allele did not cause a HIR higher than the spontaneous occurrence of haploids observed in normal non-inducer maize lines (Chase 1969). Actually, *qhir12* significantly decreased HIR in the presence of the inducer allele at *qhir11* but significantly increased HIR in the presence of the non-inducer allele at *qhir11*. In both cases, the significant differences due to the *qhir12* allele were not strong enough to change the overall expression of HI due to the *qhir1*1 allele, but merely modified the HIR.

A genome-wide study on 53 haploid inducers publicly available and 1,482 normal maize lines provided strong evidence that *qhir11* and *qhir12* were fixed in all the inducers and this was exclusively attributed to selection for HI (Hu et al. 2016). The *qhir11* sub-region, also found significant in the study by Hu et al. (2016), revealed two haplotypes, where the minor haplotype was shared by two non-inducer lines, which did not have HI ability. Additionally, Hu et al. (2016) identified *qhir12* as the most probable genomic segment carrying gene(s) responsible for HI, as this region had a single haplotype that was unchanged in all the inducers. In contrast, the results of our validation study clearly show that the major haplotype of *qhir11* found by Hu et al. (2016) is mandatory for HI and that the presence or absence of inducer *qhir12* did not affect the HIR significantly. Our study cannot make any inference on the effect of the minor haplotype of *qhir11* that was present only among two publically available inducers analyzed. Also, our study cannot make any specific conclusion regarding the 243 kb fine-mapped genomic region for HIR (Dong et al. 2013), as we have not studied this region in particular, but rather a larger genomic region harboring this fine-mapped region.

### Traits associated with maternal haploid induction

Various authors suggested investigating segregation distortion as a means to further fine-map the *qhir11* sub-region influencing maternal haploid induction in maize (Barret et al. 2008; Prigge et al. 2012b; Dong et al. 2013). Strong segregation distortion was reported against the haploid inducer allele in many genetic studies (Barret et al. 2008; Prigge et al. 2012b; Dong et al. 2013). Xu et al. (2013) studied segregation distortion in regard to HI and mapped a major QTL associated with segregation distortion, *sed1*, on chromosome 1, overlapping with the fine-mapped *qhir1* QTL. It is not clear yet, if segregation distortion is due to the same gene causing HI, or if another gene reducing fitness is closely linked to the gene(s) in *qhir1* causing HI. It is also not clear exactly what type of reduction in fitness is linked to HI. Barret et al. (2008) suggested impediments in male gametic transmission associated with HI, while Xu et al. (2013) proved that there is both gametic and zygotic selection responsible for segregation distortion associated with HI. Our study did not aim to distinguish whether segregation distortion was caused by the same gene responsible for HI, or by another tightly linked gene. However, we observed in this study that both HI ability and strong segregation distortion against the inducer *qhir11* allele, both of which were not observed for *qhir12*. For *qhir12*, the observed segregation distortion was significantly smaller, and in the opposite direction, favoring the inducer allele, while a much smaller effect was found on the HIR.

In addition to SD, high maternal HI also is strongly associated with the formation of defective kernels, including embryo and endosperm abortion (Xu et al. 2013) and reduced seed set (Satarova and Cherchel 2010). Similar to its effects on SD, the *qhir11* sub-region in our study strongly increased kernel abortion while *qhir12* had negligible effect on this. It is possible that the same gene(s) conditioning the HIR or another tightly linked gene within the *qhir11* region can condition kernel abortion. One hypothesis for this relationship is that one of the sperm cells from the inducer pollen could be defective while the other sperm cell is normal (Geiger 2009). When the defective sperm cell fertilizes the central cell, endosperm abortion can result, and when the defective sperm cell fertilizes the egg cell, a haploid embryo or aborted embryo can result. This hypothesis was supported by the occurrence of morphologically different sperm cells (Bylich and Chalyk 1996), aneuploid microsporocytes which may produce aneuploid sperm cells (Chalyk et al. 2003), and an increase in heterofertilization when haploid inducer pollen is used (Kraptchev et al. 2003; Rotarenco and Eder 2003). Another hypothesis involves epigenetic, dosage-dependent modification of the chromosomes exerted by the *sed1* locus which overlaps with the *qhir1* locus resulting in incomplete penetrance of the *sed1*/*qhir1* locus (Xu et al. 2013). It was proposed that expression of the *sed1* locus can differ between the pollen grains resulting in some pollen grains having strong epigenetic modification while others are less modified. A strong modification of the sperm cell chromosomes may lead to kernel abortion or haploid formation while less epigenetically modified pollen leads to normal kernel formation. Further studies are required to understand the exact mechanism(s) behind kernel abortion associated with HI, for which cloning the gene(s) underlying these loci could be critical.

### Putative candidate genes in the *qhir11* physical interval

The physical interval of *qhir11* in the B73 genome sequence (V2) has 13 protein-coding genes annotated by the MAKER gene annotation pipeline (Cantarel et al. 2008) as available in http://ensembl.gramene.org/Zea_mays (Suppl. Table 2). Out of these genes, 11 are predicted to have protein domains with known functions. Among these, gene Zm00001d029411 is predicted to have a protein which falls into the CULLIN family of ubiquitin ligases.

CULLIN-dependent ubiquitin ligases form a class of structurally related multi-subunit enzymes that control the rapid and selective degradation of important regulatory proteins involved in cell cycle progression and development (Thomann et al. 2005). In mice, knocking out a cullin-RING ubiquitin ligase leads to infertile male mice, due to fewer numbers of mature spermatozoa, most of which exhibit morphological defects, rendering them immotile and unable to fertilize eggs. In addition to the morphological abnormalities, chromosomal defects were also observed which may also contribute to infertility (Yin et al. 2011). The gene Zm00001d029411 in B73 had maximum similarity to AtCUL1 in *Arabidopsis thaliana*, based on a BLAST N alignment (*E* = 0.0012). CUL1 forms part of the SCF (SKP1-CUL1-F-box) complex in plants and animals, where SCF-dependant ubiquitylation plays a critical role in the control of the cell cycle (Thomann et al. 2005). Consistent with such a role, *Arabidopsis* cul1 loss-of function mutants arrest early during embryogenesis at the zygote stage (Shen et al. 2002). Genetic analysis also indicated a reduction in transmission of the *atcul1* mutation through both male and female gametes. Considering the specific roles the protein domain plays in cell cycle and gametophyte development and transmission, this gene could be an interesting putative candidate gene for HI ability. Several recent studies indicate that manipulation of Centromere Histone CENH3 could lead to in vivo haploid induction in *Arabidopsis* (Ravi and Chan 2010; Seymour et al. 2012; Ravi et al. 2014), and in maize—(Kelliher et al. 2016). However, native CENH3 may not have any role in in vivo HI using maternal haploid inducers in maize. CENH3 is localized on chromosome 6.06 (Prigge et al. 2012b) and no mapping study has so far detected a major QTL for HI in this region. Also, study by Kelliher et al. (2016) showed that altered CENH3 when introduced into maize showed a maximum of 3.6% HIR, which is significantly lower than the high HIR (~10% or more) obtained using the improved maternal haploid inducers (Röber et al. 2005; Prigge et al. 2012a; Chaikam et al. 2016). Our study also showed that none of the annotated genes at *qhir11* are related to CENH3. Therefore, cloning of the gene(s) responsible for maternal haploid induction, underlying *qhir11*, may provide a better insight into the genetic mechanism underlying gynogenesis in maize. It also needs to be explored whether CENH3-mediated HI can be synergistic to the *qhir1* mediated HI in maize.

## Conclusions

In this study, the *qhir1* region was genetically delineated, and the haploid induction ability of *qhir11* and *qhir12* sub-regions was dissected through analysis of recombinants from a large F2 population derived from a non-inducer x haploid inducer cross. The study clearly revealed that *qhir11* is the only sub-region with a strong effect on HIR, whereas *qhir12* had a negligible effect on HIR, in contrast to the hypothesis of Hu et al. (2016) based on a selective sweep based GWAS approach. Furthermore, our study proved that *qhir11* is more strongly associated than *qhir12* with segregation distortion and kernel abortion, two traits that are associated with maternal haploid induction. The results of this study give direction in further fine mapping and cloning of the gene/s underlying *qhir1*. The molecular markers delineating *qhir11* can be used for more efficient development of new inducer lines adapted to diverse agro climatic zones using marker assisted selection.

### Author’s note

When this publication was in production, three articles (Kelliher et al. 2017; Gilles et al. 2017; Liu et al. 2017) were published about cloning the gene underlying qhir1 QTL that codes for a sperm specific phospholipase and triggers haploid induction.

### Author contribution statement

AEM, SKN, VC and PMB designed the experiments. VC, ML and LLA coordinated the field trials and phenotyping. SKN and VC coordinated the sample collection, DNA extraction and genotyping. WM, SKN, VC and AEM analyzed the data. SKN, VC and WM wrote the manuscript. AEM and PMB edited the manuscript.

## Electronic supplementary material

Below is the link to the electronic supplementary material.

Suppl. Fig 1: Effects of AAbb and aaBB genotypes (a) and AABB and aabb (b) on kernel abortion in selfed F3 ears (TIF 2581 KB)

Supplementary material 2 (DOCX 19 KB)

## Abbreviations

DH: Doubled haploid
KAR: Kernel abortion rate
KAS: Kernel abortion score
HI: Haploid induction
HIR: Haploid induction rate
SD: Segregation distortion
GWAS: Genome wide association study
