# Supplementary figures and images for: Dissection of a major QTL qhir1 conferring maternal haploid induction ability in maize
Source: Theor Appl Genet. 2017 Mar 18;130(6):1113–22. doi: 10.1007/s00122-017-2873-9 (PMC5440511; doi:10.1007/s00122-017-2873-9)

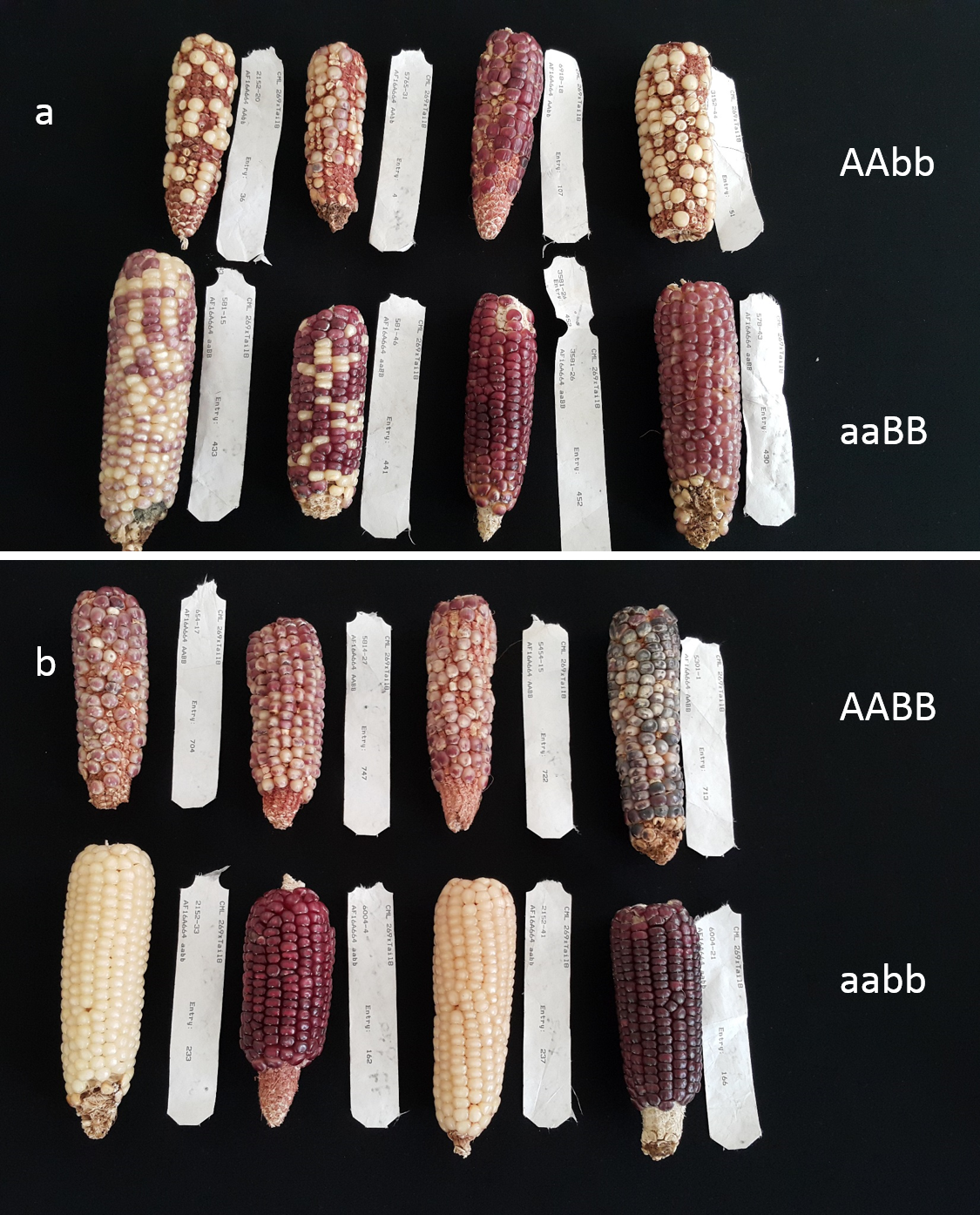

Supplement: Supplementary file 1 — Suppl. Fig 1: Effects of AAbb and aaBB genotypes (a) and AABB and aabb (b) on kernel abortion in selfed F3 ears (TIF 2581 KB) [file 122_2017_2873_MOESM1_ESM.tif]
