# Supplementary material for: Dissection of a major QTL qhir1 conferring maternal haploid induction ability in maize
Source: Theor Appl Genet. 2017 Mar 18;130(6):1113–22. doi: 10.1007/s00122-017-2873-9 (PMC5440511; doi:10.1007/s00122-017-2873-9)
Supplement: Supplementary file 2 — Supplementary material 2 (DOCX 19 KB) [file 122_2017_2873_MOESM2_ESM.docx]

**Dissection of a major QTL *qhir1* conferring maternal haploid induction ability in maize**

**Sudha Nair^1#^, Willem Molenaar^2^, Albrecht E Melchinger^2^, Prasanna M Boddupalli^3^, Leocadio Martinez^4^, Luis Antonio Lopez ^4^ and Vijay Chaikam^3*#^**

**Journal:** Theoretical and Applied Genetics

**Affiliations and address of the authors**

1: International Maize and Wheat Improvement Center (CIMMYT), ICRISAT campus, Patancheru, Greater Hyderabad 502324, India

2: Institute of Plant Breeding, Seed Science and Population Genetics, University of Hohenheim, D-70593 Stuttgart, Germany

3: International Maize and Wheat Improvement Center (CIMMYT), ICRAF campus, UN Avenue, Gigiri, P.O.Box 1041–00621, Nairobi, Kenya

4: International Maize and Wheat Improvement Center (CIMMYT), Apdo. Postal 6-641

06600, Mexico D.F, Mexico

# The first and corresponding authors contributed equally to the work.

***Corresponding author:** Vijay Chaikam

**E mail:** v.chaikam@cgiar.org

**Supplementary Tables**

**Suppl. Table 1** SNP markers used to genetically delimit sub-regions *qhir11* and *qhir12*

| SNP | Physical position B73 AGPV2 | Context sequence |
| --- | --- | --- |
| *qhir11* |  |  |
| PZE-101081177 | 68,134,633 | TTCTTGGGGATTATCTTGAGCTTTTGCTTGATGACCCCATTCATGATGCC[A/G]TCAATGGTGTTGGAGCCAGGCTGGGAGTCCTGGCCGTACGTCGAGTACTT |
| PZE0166290049 | 68,136,529 | TCAGGTAGCTAGACCGTGCTCCCGTAATGAAGCACAGAGGCCGGATCATG[C/T]CCTCCTGTGCCGAAGCCGAGAAATTGTGGTGGTGTGAGGAGGGAAAGAGA |
| PZE0166357949 | 68,179,267 | GATTCGATGTTTATTTGCTGTTGTGCGATCCACATCCGGAATGGATGGAA[A/G]GGACGTTGTGATGTTTGATGAAGTGGATACAATAAGGCCAGAAGCATACA |
| PZE-101081269 | 68,241,700 | AGAGCGTTCTTCAGAGGCGTGCTCTTGGCCTGCAAATTGGCGCAGCAGAT[A/C]GGAAAGAGCTGAGTATGTTAGCGCACGGCGTACGTAGGCAGCCATTCGCA |
| PZE-101081484 | 68,558,721 | GTCTATATTCAGAGAGACCACAACTGACCTCTAGGATCAAATGAACTGGT[A/G]GCCTTAAGGTGTGCTGCTTTCTGTAGTTCTTTCATTGATTATCAAGTCTG |
| SYN25793 | 68,670,617 | GGAGCTTGAGCTGGTTGAGGCTGGCTGTTCGACAAGACCTAACTCATCCCTGTACTGAGA[A/G]ACGGCTGCATTTGGGAAGGTTTTGTTGCCGTCTGATGGCCCAGCAGAAGCAGAGGACTCC |
| *qhir12* |  |  |
| SYN4966 | 71,795,509 | ACCCAACCGGGAGAAGCAGAGGGAGAGCAGTCACCAAACCAAAACCGCTCTCGAGTCTCC[A/C]TCCATTCTCTCCGCTCATCCGCAGCTCTCGAGTCTCCAGCTCTAGCTAGCCTTAGCCTCG |
| PZE-101083767 | 71,607,659 | GGTTGGGCCAGGCTATGAACTAACACGAGATATGTAACCGTGTCTGGGTC[A/G]ACAAGCTCAAGCAGCTCGCGCTAGAGTGGGCCGGGCCGATGTTTGCTTCT |
| SYN26730 | 73,637,089 | TTCCAGGTTGATGCAGACCTTAGAGCAGTTTCCTTCACTCTTGACCCGAAGGAGAGAAAA[A/G]GGATACAAAAAGAAACACTTTCTGCTCTGTTTGAGACTTACTTCCGCATTCTGAAGCATA |
| PZA00714.1 | 75,768,235 | AACCASTGTNCGAAATCATCTTCAGATGCAACTGACGTGTCATTGCTTGGACTCATCATC[A/G]TCATCCGAACCGTGATCGTCATCAGAAGACTCCACTCTGTTAGGTTCATCACCACCGTTA |
| PZE-101084367 | 72,842,586 | TGTTACGCTAGGTGTCCCGATCTTAGAAGCACGAATGCTGCATGCCTGCA[A/G]ATCACGCTGTAGTTGAATTTCAAGTGGGTTTTTTTTTTTGCCCGCTCTTA |
| SYN16337 | 73,510,051 | TGGAGAAAAGATCTAGACGTGTTTATGAGCACTCCTTTCGCAAGTCCAAGCATTGGAGAG[A/G]AGATCTAGACATGTTTATGAGCTGCTCTTGATCTCGTCTTAGGTTTACAAATATGGAAAG |
| PZE101085336 | 74,711,007 | TGCGCGCGCACCATAATAAGTATTGTGTGTACGTATATATCCGCATCTGC[A/G]GTGTTTGTGTCATATATAAAATAATCGTCTGCGTGCGCTATATAATATCT |
| PZE-101085916 | 75,898,561 | GATGAATGGAGTGACGATGAGGCCTAGAGATACTGTTACATGTTTGTCGC[A/C]TTTGCAAAAGCACACATGACACATGCATGCTGTTTGTTGTTTAATATTGT |

**Suppl. Table 2** Protein coding genes annotated by the MAKER gene annotation pipeline in http://ensembl.gramene.org/Zea_mays in *qhir11* physical interval

| Gene name | Functional domain |
| --- | --- |
| Zm00001d029410 | carboxy peptidase |
| Zm00001d029412 | Patatin/Phospholipase-A2 realted |
| Zm00001d029411 | cullin family |
| Zm00001d029413 | histidine phosphotase superfamily |
| Zm00001d029414 | Unknown |
| Zm00001d029416 | Thiolase |
| Zm00001d029415 | Pyruvate carboxyl transferase |
| Zm00001d029417 | P-loop containing nucleoside triphosphate hydrolase |
| Zm00001d029419 | PPM type phosphatase |
| Zm00001d029418 | Ribosomal protein L37e/L37ae |
| Zm00001d029420 | WEB family |
| Zm00001d029422 | Reverse transcriptase domain |
| Zm00001d029421 | unknown |
